# Decreased TNF Levels and Improved Retinal Ganglion Cell Survival in MMP-2 Null Mice Suggest a Role for MMP-2 as TNF Sheddase

**DOI:** 10.1155/2015/108617

**Published:** 2015-09-15

**Authors:** Lies De Groef, Manuel Salinas-Navarro, Griet Van Imschoot, Claude Libert, Roosmarijn E. Vandenbroucke, Lieve Moons

**Affiliations:** ^1^Laboratory of Neural Circuit Development and Regeneration, Animal Physiology and Neurobiology Section, Department of Biology, KU Leuven, Naamsestraat 61, 3000 Leuven, Belgium; ^2^Inflammation Research Center, VIB, FSVM Building, Technologiepark 927, 9052 Ghent, Belgium; ^3^Department of Biomedical Molecular Biology, Ghent University, FSVM Building, Technologiepark 927, 9052 Ghent, Belgium

## Abstract

Matrix metalloproteinases (MMPs) have been designated as both friend and foe in the central nervous system (CNS): while being involved in many neurodegenerative and neuroinflammatory diseases, their actions appear to be indispensable to a healthy CNS. Pathological conditions in the CNS are therefore often related to imbalanced MMP activities and disturbances of the complex MMP-dependent protease network. Likewise, in the retina, various studies in animal models and human patients suggested MMPs to be involved in glaucoma. In this study, we sought to determine the spatiotemporal expression profile of MMP-2 in the excitotoxic retina and to unravel its role during glaucoma pathogenesis. We reveal that intravitreal NMDA injection induces MMP-2 expression to be upregulated in the Müller glia. Moreover, MMP-2 null mice display attenuated retinal ganglion cell death upon excitotoxic insult to the retina, which is accompanied by normal glial reactivity, yet reduced TNF levels. Hence, we propose a novel *in vivo* function for MMP-2, as an activating sheddase of tumor necrosis factor (TNF). Given the pivotal role of TNF as a proinflammatory cytokine and neurodegeneration-exacerbating mediator, these findings generate important novel insights into the pathological processes contributing to glaucomatous neurodegeneration and into the interplay of neuroinflammation and neurodegeneration in the CNS.

## 1. Introduction

The subfamily of the gelatinases, consisting of matrix metalloproteinase-2 (MMP-2, gelatinase A, 72 kDa type IV collagenase) and matrix metalloproteinase-9 (MMP-9, gelatinase B, 92 kDa type IV collagenase), is the most extensively studied subfamily of matrix metalloproteinases (MMPs). In the central nervous system (CNS), their involvement in Alzheimer's disease, multiple sclerosis, amyotrophic lateral sclerosis, epilepsy, meningitis, and so forth has been well studied, leading to insights into their role in pathophysiological processes such as blood-brain barrier disruption, neuroinflammation, demyelination, neuronal cell death, and brain edema [1–4]. Unfortunately, this has somewhat overshadowed their potential as beneficial modulators of CNS development, plasticity, and repair [5].

Also in the eye, MMPs are needed for the development and physiology of the lens, cornea, retina, sclera, and trabecular meshwork, but unrestrained MMP activity might underlie a number of ocular pathologies and retinal degenerations, including glaucoma [6–11]. In the retina, increased MMP-9 activity in the ganglion cell layer (GCL), expressed by either reactive astrocytes [10, 11] or retinal ganglion cells (RGCs) [12, 13], plays a key role in the promotion of RGC death. Increased MMP-9 expression/activity in the retina was reported upon induction of ischemia-reperfusion injury [11, 13–16], N-methyl-D-aspartic acid (NMDA) and kainic acid-mediated excitotoxicity [10, 12], optic nerve transection [17], and ocular hypertension [18] in rodents. Moreover, this increase in MMP-9 activity positively correlated with RGC death, by promoting laminin degradation and detachment-induced apoptosis of RGCs [13, 18, 19]. The ultimate confirmation of this detrimental role of MMP-9 was seen in MMP-9 null mice, which were protected from laminin degradation and RGC death after optic nerve ligation [14]. In contrast to MMP-9, virtually nothing is known about the role of MMP-2 in the healthy nor the glaucomatous retina. MMP-2 has been reported being expressed by RGCs, Müller glia, and astrocytes in the mouse retina and by RGC somata and axons in primates [10, 20, 21], yet it is still under debate whether MMP-2 becomes upregulated upon glaucomatous damage to the retina or whether its expression levels remain unchanged [10, 12, 15–17, 20]. Moreover, in contrast to MMP-9, MMP-2 deficiency did not protect against RGC death in the optic nerve ligation glaucoma model [14].

Here, we describe the spatiotemporal expression of the gelatinases MMP-2 and MMP-9 in the retina of mice that received an intravitreal injection of the glutamate analogue NMDA in order to induce RGC death. This experimental glaucoma model mimics excitotoxic damage to the retina [22–27], as glutamate is the predominant excitatory neurotransmitter in the CNS and overstimulation of its receptors leads to excessive Ca^2+^ influx and activation of apoptotic signaling cascades. This excitotoxic injury is common to many neurological disorders [28], including glaucoma [29–31], in which the retinal neurodegeneration ties to the exquisite sensitivity of the RGCs to glutamate and the glutamate analogue NMDA. Next, we focused on MMP-2 and explored its contribution to RGC death following NMDA-induced excitotoxic retinal injury. Therefore, we investigated differential susceptibility to NMDA-induced RGC degeneration in wild type and MMP-2 null mice and explored three putative mechanisms by which MMP-2 could contribute to RGC death: (1) laminin degradation leading to detachment-induced apoptosis, (2) glial reactivity, and (3) potentiation of glutamate excitotoxicity by tumor necrosis factor (TNF).

## 2. Methodology

### 2.1. Experimental Animals

All studies were conducted in compliance with the European Communities Council Directive of 22 September 2010 (2010/63/EU) and the Belgian legislation (KB of 29 May 2013) and were approved by the KU Leuven institutional ethical committee. Adult (2-3 months) wild type, MMP-2 null [32], and MMP-9 null mice [33], all with a C57Bl6/N genetic background, were obtained from the university breeding colony. Wild type, MMP-2 null, and MMP-9 null mice were genotyped to confirm homozygosity, as described by [32, 33]. Animals were kept under a 12/12 light-dark cycle and had* ad libitum* access to food and water.

### 2.2. Surgical Procedures

All surgical procedures were performed under general anesthesia (i.p. 75 mg/kg body weight ketamine, Anesketin, Eurovet; i.p. 1 mg/kg medetomidine, Domitor, Pfizer). After the procedure, anesthesia was reversed by means of atipamezole (i.p. 1 mg/kg, Antisedan, Pfizer), and antibiotic ointment (tobramycin 3 mg/g, Tobrex, Alcon) was applied to avoid corneal desiccation and infection of the eye. In order to exclude contralateral effects, which have been reported, for example, for GFAP expression and microglia reactivity [34–36], untreated eyes from a separate cohort of untreated animals served as controls.

#### 2.2.1. Intravitreal NMDA Injection

Intravitreal injection was performed as described [24]. Briefly, NMDA (20 mM or 4.0 mM in phosphate-buffered saline (PBS); Sigma-Aldrich) was injected into the superior quadrant of the right eye using a glass capillary with a 50–70 *μ*m outer diameter, connected to a Hamilton syringe. The needle tip was inserted into the vitreous chamber 1 mm behind the limbus under a 45-degree angle to avoid damage to the lens, and a total volume of 2 *μ*L (0.5 *μ*L/sec) was injected. In addition to general anesthesia, eye drops with topical anesthesia (oxybuprocaine 0.4%, Unicaïne, Théa) were given. Animals were sacrificed at 1 day postinjury (dpi).

#### 2.2.2. Optic Nerve Crush

Optic nerve crush (ONC) was performed as described [37]. Briefly, an incision was made in the skin overlying the superior orbital rim, the superoexternal orbital contents were dissected, and the superior and external rectus muscles were transected. The exposed optic nerve was then crushed 1 mm from the globe with a watchmaker's forceps for 10 sec. Fundoscopy was performed before and after the procedure to assess integrity of retinal perfusion. Animals were sacrificed at 4 dpi.

### 2.3. Immunohistochemistry

#### 2.3.1. MMP Immunostaining on Cryosections

Mice were deeply anaesthetized (i.p. 30 mg/kg sodium pentobarbital, Nembutal, Ceva) and perfused transcardially with 4% PFA. Eyes were dissected and postfixed overnight in 4% PFA, and the cornea and lens were removed. The remainder posterior segment of the eye was cryoprotected in a 10%-20%-30% sucrose series (in PBS) and embedded in Tissue-Tek optimal cutting temperature medium (Sakura Finetek) to make transverse cryosections (10 *μ*m).

Antigen retrieval was performed by heating the sections in citrate buffer (10 mM [pH 6.0]) for 20 min at 95°C, followed by a 20 min cooling down. Next, sections were incubated for 20 min in 0.3% hydrogen peroxidase (in methanol) to saturate endogenous peroxidases and subjected to a 1 h blocking step with 20% preimmune serum. Sections were incubated overnight with the primary antibody at room temperature. Based on a comparison of antibody specificity by De Groef et al. [38], the following antibodies were used: rabbit anti-MMP-2 (Millipore, ab19167) (1 : 300) and mouse anti-MMP-9 (Abcam, ab58803) (1 : 300). Secondary IgG antibodies were conjugated to biotin (Dako) (1 : 300) and applied for 45 min, followed by 30 min incubation with streptavidin-horse radish peroxidase (HRP) (Perkin-Elmer) (1 : 100). Finally, fluorescein isothiocyanate tyramide signal amplification was performed according to the manufacturer's instructions (Perkin-Elmer). Sections were rinsed with Tris-buffered saline (TBS) in between steps, and preimmune serum and antibodies were diluted in 0.5% blocking solution (Perkin-Elmer). 4′,6-Diamidino-2-phenylindole (DAPI) (1 *μ*g/mL in PBS, AppliChem) was used as a fluorescent nuclear counterstaining and sections were mounted using Mowiol antifading medium (10% Mowiol 4–88 (Sigma-Aldrich), 40% glycerol, and 0.1% 1,4-diazabicyclo-[2,2, 2]-octane in 0.2 M Tris-HCl [pH 8.5]). All images were taken with an inverted confocal microscope (FV1000, Olympus) and were processed with FluoViewer (Olympus) and Photoshop CS2 (Adobe) software.

#### 2.3.2. Brn3a Immunostaining on Retinal Flat Mounts

Mice were deeply anaesthetized (i.p. 30 mg/kg sodium pentobarbital, Nembutal, Ceva) and sacrificed by cervical dislocation. Next, eyes were dissected and fixed for 1 h in 4% phosphate-buffered paraformaldehyde (PFA). The retina was dissected and flat-mounted and again fixed for 1 h in 4% PFA. Prior to immunohistochemistry, retinal flat mounts were rinsed in PBS with 0.5% Triton X-100 for 3 times for 10 min.

Retinas were frozen for 15 min at −80°C, before applying the primary goat anti-Brn3a antibody (Santa Cruz, C-20, sc-31984) (1 : 750), which has been shown to selectively label RGCs [39, 40]. On the next day, a secondary rabbit anti-goat IgG antibody conjugated to Alexa fluorophore-488 (Life Technologies) (1 : 500) was applied for 2 h. Retinal flat mounts were rinsed with PBS with 0.5% Triton X-100 in between steps, and all antibodies were diluted in PBS containing 2% Triton X-100 and 2% rabbit preimmune serum. Mosaic z-stack images of the entire retina were taken with a multiphoton microscope (BX61WI, Olympus), equipped with a MaiTai HP DeepSee laser (690–1020 nm, Spectra Physics) and FluoViewer 4.0 software (Olympus).

### 2.4. Gelatin Gel Zymography

Mice were deeply anaesthetized (i.p. 30 mg/kg sodium pentobarbital, Nembutal, Ceva) and sacrificed by cervical dislocation. Retinas were quickly dissected and homogenized in ice-cold lysis buffer (50 mM Tris-HCl [pH 7.6], 5 mM CaCl_2_, 150 mM NaCl, 0.05% Brij-35 (Sigma-Aldrich), 1% Triton X-100, and 100 *μ*M phenylmethylsulfonyl fluoride), supplemented with an EDTA-free proteinase inhibitor cocktail (Roche). Upon homogenization, samples were centrifuged, supernatant was collected, and protein concentrations were measured with Qubit fluorometric quantitation (Life Technologies).

Aliquots containing 120 *μ*g of total proteins were incubated with 50 *μ*L gelatin-conjugated Sepharose beads (gelatin Sepharose 4B, GE Healthcare) in equilibrating buffer (0.5 M NaCl, 10 mM CaCl_2_, and 0.01% Tween-20 in TBS) for 20 min at room temperature, for affinity precipitation. Next, the beads were rinsed twice with TBS containing 0.5 M NaCl, 10 mM CaCl_2_, and 0.05% Tween-20 and once with TBS containing 10 mM CaCl_2_ and 0.05% Tween-20. Finally, gelatinases were eluted with 20 *μ*L zymogram loading buffer (Novex Tris Glycine SDS Sample Buffer, Life Technologies) and loaded on a 10% gelatin gel (Novex, Life Technologies) for electrophoresis. Gels were incubated in 2.5% Triton X (in water) for 30 minutes and developed for 2 days at 37°C in TBS containing 10 mM CaCl_2_ and 1.25% Triton X-100. After staining with Coomassie blue (0.5% in a mixture of 9 : 9 : 2 methanol, water, and acetic acid) for 3 h, gels were destained for 2 h (in a mixture of 9 : 9 : 2 ethanol, water, and acetic acid) and imaged with the ChemiDoc MP Imaging System (BioRad). Quantification was performed with Image Lab 4.1 (BioRad).

### 2.5. Western Blotting

Mice were deeply anaesthetized (i.p. 30 mg/kg sodium pentobarbital, Nembutal, Ceva) and sacrificed by cervical dislocation. Retinas were quickly dissected and homogenized in ice-cold lysis buffer (50 mM Tris-HCl [pH 7.5], 10 mM CaCl_2_, 150 mM NaCl, 0.05% Brij-35 (Sigma), and 1% Triton X-100), supplemented with an EDTA-free proteinase inhibitor cocktail (Roche). Upon homogenization, samples were centrifuged, supernatant was collected, and protein concentrations were measured with Qubit fluorometric quantitation (Life Technologies).

Retinal homogenates (25 *μ*g) were loaded on 4–12% SDS-PAGE and transferred onto a polyvinylidene fluoride membrane. After 2 h of blocking with 5% Amersham ECL Blocking Agent (GE Healthcare), membranes were incubated overnight at room temperature with the primary antibody. The following antibodies were used: rabbit anti-laminin (Sigma-Aldrich, L9393) (1 : 1000) and rabbit anti-glial fibrillary acidic protein (GFAP) (Dako, Z0334) (1 : 20000). On the next day, membranes were incubated for 45 min with HRP-labeled secondary goat anti-rabbit IgG antibody (1 : 20000 for laminin, 1 : 100000 for GFAP) and protein bands were visualized with the ChemiDoc MP Imaging System (BioRad), using a luminol-based enhanced chemiluminescence (ECL) kit (Thermo Scientific). All antibodies were diluted in 5% Amersham ECL Blocking Agent (GE Healthcare) (in TBS). Data were normalized using a total protein stain with Coomassie blue and quantification was performed with Image Lab 4.1 (BioRad).

### 2.6. TNF Immunoassay

Mice were deeply anaesthetized (i.p. 30 mg/kg sodium pentobarbital, Nembutal, Ceva) and sacrificed by cervical dislocation. Retinas were quickly dissected and homogenized in ice-cold PBS, supplemented with an EDTA-free proteinase inhibitor cocktail (Roche). Upon homogenization, samples were centrifuged, supernatant was collected, and protein concentrations were measured with the Pierce BCA protein assay kit (Life Technologies).

Quantification of TNF in retinal homogenates was performed using the Bio-Plex cytokine immunoassay (BioRad), according to the manufacturer's instructions. For each sample, 50 *μ*g of total protein was used.

### 2.7. Quantification of RGC Survival

RGC density (number of RGCs/mm²) was evaluated on entire retinal flat mounts after immunostaining for Brn3a and automatically computed using Fiji software [41] and an in-house made macro. Briefly, a rolling ball background subtraction was used and, after local thresholding, Brn3a immunopositive RGCs were automatically counted, based on selection criteria for size and shape (circularity). The retinal flat mount was manually outlined and its surface was computed, to yield the total number of RGCs over the total surface of the retina.

### 2.8. Statistics

Normal distribution was verified using a Kolmogorov-Smirnov test and parallel equal variance between groups was tested. Outliers were identified and excluded, based on Grubbs test. Zymography data were analyzed using a one-way ANOVA with Dunnett's* post hoc* multiple comparisons test. Western blot data for laminin and GFAP were analyzed using a two-way ANOVA. RGC survival was analyzed using Student's *t*-test. TNF levels were analyzed using both two-way ANOVA and Student's *t*-test. A probability level (*α*-level was set to 0.05) of <0.05 was accepted as statistically significant (^*∗*^ *p* < 0.05, ^*∗∗*^ *p* < 0.01, and ^*∗∗∗*^ *p* < 0.005). All data are presented as mean ± SEM. Statistical analyses were performed using GraphPad Prism 6 (GraphPad Software).

## 3. Results

### 3.1. Spatiotemporal Expression Patterns of MMP-2 and MMP-9 in the Excitotoxic Mouse Retina

**Figure 1:**
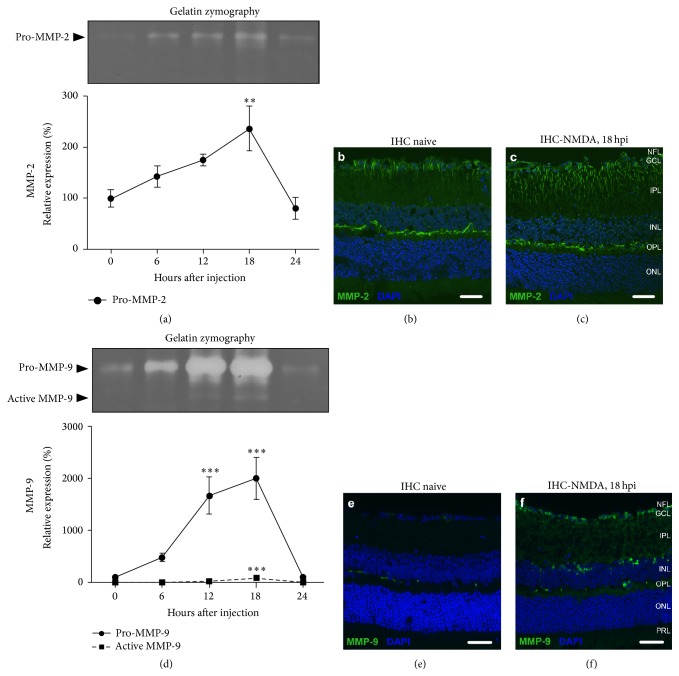
Expression profiles of MMP-2 and MMP-9 in the excitotoxic mouse retina. (a) Upon excitotoxic injury to the retina, pro-MMP-2 expression, as revealed by gelatin zymography, was upregulated, reaching maximum expression at 18 hpi and returning to baseline levels by 24 hpi. (b) Immunohistochemical (IHC) staining for MMP-2 (green) in the naive retina. (c) Immunostaining for MMP-2 revealed that the increased MMP-2 expression at 18 hpi localized to the activated astrocytes and/or radial processes of the Müller glia. (d) Gelatin zymography disclosed that, upon excitotoxic injury to the retina, pro-MMP-9 steeply increased to reach a maximum at 18 hpi, accompanied by a lower peak of activated MMP-9. At 24 hpi, both mature and pro-MMP-9 levels had returned to baseline. (e) Immunostaining for MMP-9 (green) in the naive retina. (f) Upon excitotoxic injury to the retina, an increased number of MMP-9^+^ microglia and RGCs, and augmented MMP-9 immunoreactivity in the plexiform layers were observed. Scale bars, 20 *μ*m.

Expression levels of MMP-2 and MMP-9 were studied in the retina under physiological conditions and within the first 24 h after intravitreal administration of NMDA. In the naive retina, gelatin zymography revealed that both gelatinases were present in their proform and no mature MMP-2 or MMP-9 was seen. Upon excitotoxic injury to the retina, the expression of the proform of both gelatinases was upregulated. For MMP-9, this resulted in a 20-fold upregulation of pro-MMP-9 expression and a significant rise in mature MMP-9 at 18 hours postinjection (hpi) (Figure 1(d)). For MMP-2, expression of the proform was nearly 2.5 times upregulated at 18 hpi, yet mature MMP-2 remained below detection levels (Figure 1(a)). Remarkably, the expression of mature MMP-9, pro-MMP-9, and pro-MMP-2 had returned to baseline levels by 24 hpi.

Immunostaining for MMP-2, at its peak of expression (18 hpi), revealed an increased labeling of activated astrocytes and/or Müller glia as compared to the naive retina (Figures 1(b)-1(c)) [38]. On MMP-9 immunostainings, an increased number of MMP-9 immunopositive microglia near the borders of the inner nuclear layer (INL) were seen [38] and augmented MMP-9 immunoreactivity in the plexiform layers. Furthermore, while in the naive retina RGCs were only sporadically labeled for MMP-9 [38], the majority of RGCs were MMP-9 immunopositive in the excitotoxic retina (Figures 1(e)-1(f)).

### 3.2. MMP-2 Deficiency Attenuates Excitotoxic RGC Death

**Figure 2:**
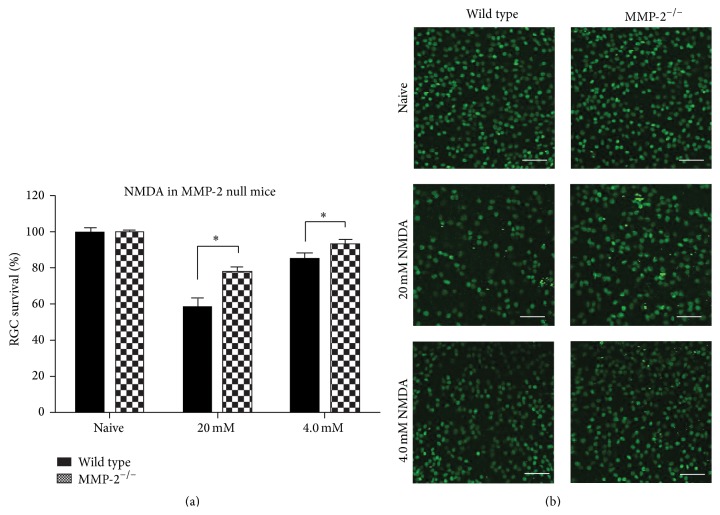
MMP-2 null mice are less susceptible to NMDA-induced RGC death. (a) Quantification of the number of Brn3a^+^ RGCs in two independent experiments (20 mM or 4.0 mM NMDA), during which different severities of RGC death were evoked, revealed that RGC death is attenuated in MMP-2 null mice (mean ± SEM, *N* ≥ 5, Student's *t*-test). (b) RGC survival was assessed at 24 hpi by means of Brn3a immunolabeling of viable RGCs. Scale bar, 100 *μ*m.

Given the upregulated expression of MMP-2 upon excitotoxic retinal damage seen in this study, we next investigated whether MMP-2 null mice also displayed altered sensitivity to experimentally induced RGC death. Two independent experiments, with varying severities of RGC death induced by different doses of NMDA, were performed to investigate whether wild type and MMP-2 null mice are differentially susceptible to NMDA-induced RGC death. In the first high-dose experiment, wild type mice displayed 58 ± 8% RGC survival at 24 hpi, yet MMP-2 null mice proved to be more resistant to RGC death and displayed 78 ± 2% RGC survival (*p* < 0.05). The second low-dose experiment revealed 85 ± 7% RGC survival in the wild type mice and yet no significant RGC death (93 ± 7% survival) in MMP-2 null mice (*p* < 0.05) (Figure 2) (Table 1). These data thus reveal that RGC death is attenuated in MMP-2 deficient animals.

### 3.3. Potential Mechanisms Underlying Increased RGC Survival in MMP-2 Null Mice

#### 3.3.1. Laminin-Integrin Signaling

**Figure 3:**
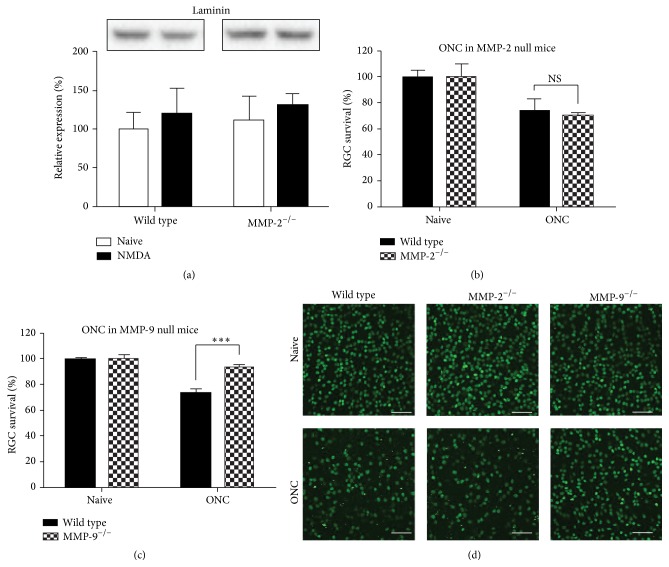
Differential mechanisms underlie RGC death in NMDA-induced excitotoxicity and optic nerve crush models. (a) Western blot for laminin (200–220 kDa) on retinal samples from wild type and MMP-2 null mice, either naive or subjected to the NMDA-induced excitotoxicity model, revealed no differences in laminin expression at 18 hpi (mean ± SEM, *N* = 3, one-way ANOVA). (b) No difference in RGC death after ONC was seen in wild type versus MMP-2 null mice (mean ± SEM, *N* ≥ 6, Student's *t*-test). (c) Four days after ONC, MMP-9 null mice revealed attenuated RGC death as compared to wild type mice (mean ± SEM, *N* ≥ 6, Student's *t*-test). (d) Representative pictures of Brn3a immunostaining, revealing differential RGC death after ONC in wild type and MMP-2 null versus MMP-9 null mice. Scale bar, 100 *μ*m.

MMP-9 null mice, in contrast to MMP-2 null mice, were reported to be protected from RGC death induced by optic nerve ligation, and this was accompanied by preservation of the laminin composition of the inner limiting membrane and laminin-integrin survival signaling in the RGCs [13, 14, 18, 19]. Given that MMP-2 is also a gelatinase and shares many substrates with MMP-9, we performed a Western blot for laminin *β*1/*γ*1, to see whether (1) laminin is degraded after NMDA injection, as was shown in the optic nerve ligation model, and whether (2) laminin is differentially present in the retina of wild type versus MMP-2 null mice. Western blot indicates, however, that intravitreal injection of NMDA did not affect laminin integrity in the retina, not in wild type nor MMP-2 null mice (*p* = 0.39) (Figure 3(a)). Notably, in line with the findings obtained in the optic nerve ligation model [14], MMP-9 null mice displayed increased RGC survival after ONC (*p* < 0.001) (Figure 3(c)), while MMP-2 mice did not (Figure 3(b)) (Table 2). Altogether, these observations suggest that RGC death might be evoked by a different mechanism in the ONC/optic nerve ligation model versus the NMDA-induced excitotoxicity model. Nevertheless, our data suggest that laminin-dependent survival signaling in RGCs does not contribute to the neuroprotective effect of MMP-2 deficiency.

#### 3.3.2. Glial Reactivity

**Figure 4:**
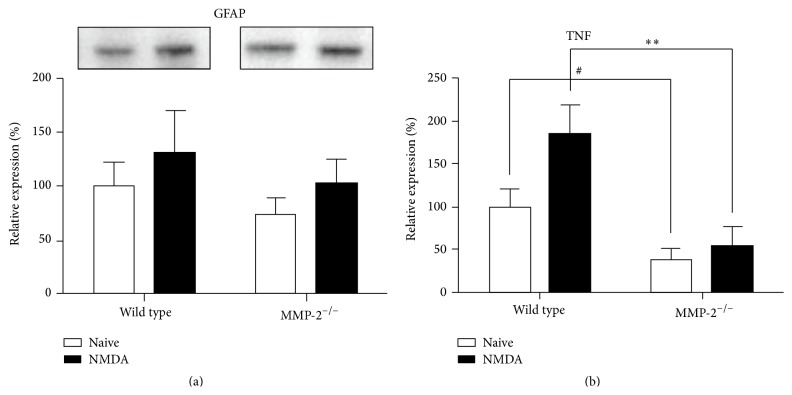
GFAP and TNF levels in wild type versus MMP-2 null mice, at baseline and 6 h after injection of NMDA. (a) Western blot revealed a trend towards increased GFAP expression upon intravitreal injection of NMDA, yet no differences between wild type and MMP-2 null mice (mean ± SEM, *N* ≥ 7, two-way ANOVA). (b) Baseline TNF levels were lower in MMP-2 null mice as compared to wild type mice (mean ± SEM, *N* ≥ 6, ^#^ *p* < 0.05, Student's *t*-test). At 6 hpi, TNF levels had increased in both wild type and MMP-2 null mice; yet MMP-2 null mice still expressed significantly less TNF than wild type mice (mean ± SEM, *N* ≥ 6, one-way ANOVA).

While neuronal cell death is the primary hallmark of neurodegeneration, it is also accompanied by reactivity of the glial cells. This gliosis is believed to serve both detrimental and beneficial functions and may have a major impact on the outcome of neurodegenerative events. The intermediate filament protein GFAP has traditionally been considered the most sensitive early indicator of reactive gliosis [42] and reflects the hypertrophic response of Müller glia in the injured retina. Given its glial expression pattern and increased expression during RGC death/retinal gliosis, which closely resembles the increase in GFAP expression that is characteristic of glial reactivity following retinal injury, we therefore investigated whether MMP-2 might be involved in reactive gliosis. Western blot analysis of GFAP expression on retinal samples isolated at 6 hpi of NMDA indicated that retinal GFAP levels tended to increase as compared to baseline GFAP expression (*p* = 0.24). Both wild type and MMP-2 null mice appeared to mount a glial response within 6 h after NMDA injection (*p* = 0.28), characterized by a ~30% increase in GFAP expression (Figure 4(a)). Taken together, glial reactivity seemed to be unaffected in MMP-2 null mice.

#### 3.3.3. TNF Signaling

The proinflammatory cytokine TNF (TNF*α*) is believed to play a central role in glaucomatous neurodegeneration [43]. Moreover, its contribution to NMDA-induced RGC death has been well described [24], as well as its inducing effects on* Mmp* gene transcription [44]. Inversely, several MMPs, including MMP-2, can activate pro-TNF [45]. Quantification of TNF by means of an immunoassay, preferentially detecting soluble TNF, revealed that baseline TNF levels were reduced by more than 60% in MMP-2 null mice. Intravitreal injection of NMDA induced a significant increase in TNF at 6 hpi (*p* < 0.05), both in wild type and MMP-2 null mice. However, while an 85% increase was seen in wild type mice, TNF levels augmented by only 18% in MMP-2 null mice, resulting in TNF levels still below the baseline levels seen in wild type mice (Figure 4(b)). Overall, TNF activation was suppressed in MMP-2 null mice, resulting in lower baseline TNF levels and reduced activation upon excitotoxic injury to the retina.

## 4. Discussion

In summary, this study reported four major findings. First, NMDA exposure caused induction of gelatinase expression/activity in the retina, including increased MMP-2 expression in Müller glia. Second, RGC degeneration induced by intravitreal injection of NMDA was diminished in MMP-2 null mice. Third, glial hypertrophy and laminin integrity were unaffected in the retina of wild type versus MMP-2 null mice exposed to NMDA. Fourth, both baseline TNF levels and the upregulation of TNF levels upon intravitreal NMDA injection were reduced in MMP-2 null mice.

### 4.1. MMP-2 Expression Is Upregulated in the Excitotoxic Mouse Retina

Available expression data and functional studies of MMP-2 in the retina are either inconclusive or lacking. Whereas some report no changes in MMP-2 activity/expression after excitotoxic injury, ischemia-reperfusion, or optic nerve transection [12, 15, 17], others described increased expression/activity in the retina at 6 h after ischemia-reperfusion injury or excitotoxicity [10, 16]. By means of gelatin zymography, we revealed a clear upregulation of pro-MMP-2 expression upon excitotoxic retinal injury that reached a maximum at 18 hpi and returned to baseline levels by 24 hpi. Furthermore, immunostaining for MMP-2 revealed that MMP-2 expression is at all times confined to the Müller glia. While both Western blot [38] and zymography results indicate that MMP-2 is present in its proform in the naive retina, it might seem surprising that no mature MMP-2 was detected upon upregulation of MMP-2 expression at 18 hpi. However, whereas gelatin zymography is believed to be the most specific technique to quantify MMP-2 expression [46], we believe that this is due to the detection limits of the gelatin zymography.

### 4.2. Glial Reactivity Is Preserved in MMP-2 Null Mice

In the healthy retina, Müller glia support neuronal function and metabolism by providing trophic support, removing metabolic waste, maintaining retinal homeostasis, and preserving the blood-retinal barrier (BRB). At the same time, they also support synaptic activity, by recycling neurotransmitters, supplying neurotransmitter precursors, and releasing gliotransmitters. In case Müller glia become activated, which happens upon virtually every pathological stimulus, they may sustain the survival of photoreceptors and neurons but may also contribute to neuronal degeneration. Indeed, glial reactivity, sometimes described as a low-grade inflammation, may help to maintain the CNS integrity, by filling gaps created by degenerating neurons, restoring damaged protective barriers, inducing the release of neurotrophic factors, clearance of excess extracellular glutamate, and so forth. However, chronic or exacerbated gliosis is also correlated with glial metabolic dysfunction, altered neuronal electrophysiology, suppression of regeneration due to glial scarring and inflammation-induced BRB dysfunction, oxidative stress, mitochondrial dysfunction, and so forth [47–49]. Taken together, neuroinflammation and neurodegeneration are intrinsically intertwined, and glial reactivity has a profound impact on the outcome of neurodegenerative processes.

Based on its expression pattern in Müller glia, we explored a potential role for MMP-2 during reactive gliosis. Notably, in contrast to our findings, MMP-2 null mice have been shown to display impaired structural and functional recovery after spinal cord injury due to increased gliosis [50]. Also, TNF has been described to reduce GFAP expression [51, 52]. Western blot experiments revealed a trend towards an increased GFAP expression at 6 hpi, suggesting that gliosis occurred in response to NDMA-induced retinal injury. Nevertheless, no difference in GFAP upregulation was found in wild type versus MMP-2 deficient mice, and no baseline differences were seen either. Wild type and MMP-2 null animals both thus initiate reactive gliosis to the same extent, at least as analyzed via intermediate filament expression.

### 4.3. Low TNF Levels and Decreased NMDA-Induced RGC Death in MMP-2 Null Mice

MMP-2 deficiency was reported to have no effect on RGC survival upon optic nerve ligation [14], a finding we confirmed in the ONC model. In contrast, our results revealed attenuated RGC death in MMP-2 null animals exposed to yet another glaucoma model, that is, excitotoxic RGC death induced by an intravitreal injection of NMDA. Strikingly, these MMP-2 null mice showed reduced levels of TNF, a proinflammatory cytokine with a well-documented neurodegeneration-exacerbating function in glaucoma [43].

While MMP-2 null animals displayed a clearly diminished TNF upregulation in response to excitotoxic retinal injury, the significant difference in baseline TNF levels suggested that already under physiological conditions TNF expression/activity was disturbed in MMP-2 deficient mice. This is likely to be related to the role of MMPs, including MMP-2, as activators of pro-TNF. Indeed, although TNF converting enzyme (TACE) is the main metalloproteinase involved in TNF activation, it has been shown that TACE and MMPs play complementary roles in shedding of the 26 kDa transmembrane precursor protein of TNF to release the soluble, biologically active 17 kDa C-terminal part from the cell membrane [44, 45, 53–55]. Interestingly, activator protein-1 (AP-1) binding site(s) in their promoter region render most* Mmp* genes responsive to changes in the amount and/or activity of corresponding transactivators, such as TNF. In addition, the* Mmp2* gene promotor also contains a functional binding site for p53, a putative mediator of TNF-induced apoptosis, and activation of pro-MMP-2 has been shown to be stimulated via TNF-nuclear factor *κ*-light-chain-enhancer of activated B cells (NF-*κ*B) signaling [56, 57]. As such, a complex regulatory feedback loop connecting TNF activity and MMP-2 expression seems to be established, which might augment the reduction in TNF shedding and RGC vulnerability in MMP-2 null mice even more.

### 4.4. Neuroinflammation and Neurodegeneration in the Retina: A Central Role for TNF

**Figure 5:**
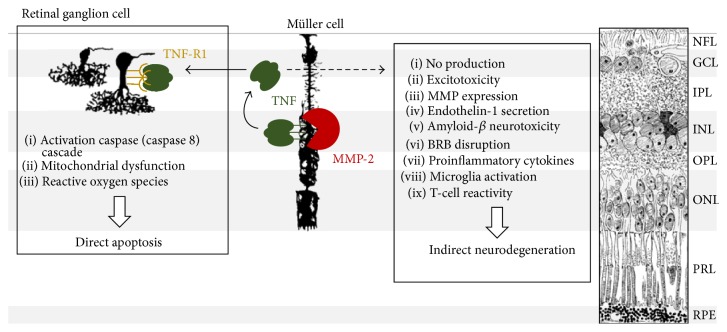
Mechanisms of TNF-induced neurodegeneration in the glaucomatous retina. In the glaucomatous retina, bioactive, soluble TNF is released by the Müller glia. Upon binding to its receptor TNF-R1 on the RGCs, TNF induces apoptosis, via activation of the caspase (caspase-8) signaling cascade, loss of mitochondrial membrane potential, and generation of reactive oxygen species. On the other hand, TNF is also involved in a complex interplay between proinflammatory, proapoptosis, and prosurvival pathways, which indirectly affect RGC survival, via nitric oxide production, modulation of neuronal excitability and excitotoxicity, expression and release of MMPs (including MMP-9), synthesis and secretion of vasoactive endothelin-1, amyloid-beta neurotoxicity, BRB disruption, and neurotoxicity of secreted cytokines and recruited microglia and T-cells. Notably, in this paper, we propose a novel* in vivo* function for MMP-2, as an activating sheddase of TNF. Drawings by Ramon Y. Cajal.

Glial production of TNF and expression of its death receptor (TNF receptor-1, TNF-R1) on RGCs and their axons are known to be upregulated in the retina and optic nerve of glaucoma patients and animals with experimentally induced glaucoma [43, 58, 59] and may induce RGC death via multiple mechanisms (Figure 5). These include direct pathways to RGC death, via binding of glial TNF to TNF-R1 on RGCs, such as caspase 8-dependent activation of the apoptosis cascade, mitochondrial dysfunction leading to the release of mitochondrial cell death mediators (cytochrome c, apoptosis inducing factor), and generation of reactive oxygen species [43, 49]. On the other hand, this potent immunomediator is also an essential signaling molecule in the interplay between neuroinflammatory and neurodegenerative events [60]. Glial reactivity and TNF activity in the glaucomatous retina can lead to nitric oxide production, modulation of neuronal excitability and excitotoxicity, release of MMP-9, BRB disruption, synthesis and secretion of vasoactive endothelin-1, amyloid-beta neurotoxicity, direct neurotoxicity of secreted cytokines, and indirect neurotoxicity by recruited microglia and T-cells [43, 49, 60]. Moreover, TNF also has a direct impact on Müller glia function, via TNF-induced release of inflammatory mediators, reactive oxygen species, and prostaglandins and even suppression of intermediate filament expression and Müller glia metabolism [60–62].

The impact of the immunomodulatory and neurotoxic actions of TNF on RGC death was illustrated in several* in vitro* studies and experimental glaucoma models [43, 63]. For instance, in case of an excitotoxic insult to the retina, NMDA-induced activation of NF-*κ*B and production of TNF in Müller glia were shown to result in RGC death. On the other hand, TNF null mice and mice that had received pharmacological anti-TNF treatment revealed remarkably reduced RGC degeneration [24]. Likewise, TNF-R1 null mice showed significantly less RGC degeneration after ONC, especially during the period correlated with glial activation and secondary neurodegeneration [64]. These data confirm the importance of TNF in glaucomatous neurodegeneration and indicate that inhibition of TNF shedding, for example, by deletion of* Mmp2*, can indeed profoundly affect the outcome of RGC degeneration.

### 4.5. Gelatinase Expression Is Upregulated in the Glaucomatous Mouse Retina and Yet Plays a Different Role Depending on the Kind of Injury

Spatiotemporal mapping of MMP-9 expression/activity revealed that MMP-9 expression and activity rose as soon as 6 hpi, reaching a maximum at 12–18 hpi, and returned to baseline levels at 24 hpi, in the excitotoxic retina. The rise in MMP-9 activity was mainly situated in the GCL, where the number of MMP-9^+^ RGCs remarkably increased, as well as in activated microglia, whose numbers also augmented in the excitotoxic retina. Accordingly, several studies in rodent models of glaucoma, induced by ischemia-reperfusion injury [11, 13–16], N-methyl-D-aspartic acid (NMDA), and kainic acid-mediated excitotoxicity [10, 12], optic nerve transection [17], and ocular hypertension [18], revealed increased MMP-9 activity in the retina, which positively correlated with RGC death. This elevated MMP-9 activity in the GCL was shown to play a key role in the promotion of RGC death, as the resulting degradation of laminin abrogated integrin-mediated survival signaling and ultimately led to a decreased expression of antiapoptotic Bcl-xL and detachment-induced apoptosis of RGCs [13, 18, 19]. Analogous to observations in MMP-9 null mice that underwent optic nerve ligation [14], we observed (partial) protection from RGC death in MMP-9 null mice 4 days after ONC, a model bearing high similarities to the optic nerve ligation model.

Remarkably, integration of the data obtained in the present study and those by Chintala et al. [14] points out that MMP-2 null mice are vulnerable to optic nerve injury-induced RGC death and yet are protected from NMDA-induced RGC death. We therefore postulate that the mechanisms underlying RGC death in the NMDA-induced glaucoma model and in the optic nerve ligation/crush models are very distinct and that MMP-2 and MMP-9 are apparently differentially involved in these different types of glaucomatous injury. Indeed, while RGC death was linked to laminin degradation in the optic nerve ligation model, the present data showed that laminin expression was preserved in the retina of mice subjected to the NMDA model.

## 5. Conclusion and Future Perspectives

In this paper, we revealed that intravitreal injection of NMDA induced a transient increase in MMP-2 in the Müller glia and that MMP-2 null mice were more resistant to NMDA-induced RGC death than wild type animals. Intriguingly, reduced baseline TNF levels and NMDA-induced TNF activation in MMP-2 null mice suggested a novel* in vivo* role for MMP-2 as a sheddase of TNF. The suppressed TNF signaling in MMP-2 null mice is likely to underlie their increased resistance to excitotoxic RGC death; however, despite ruling out glial reactivity and laminin-mediated survival signaling, we could not yet pinpoint the exact mechanism whereby TNF exerted its effects.

Interestingly, TNF has been widely recognized as an attractive therapeutic target. Multiple (pre)clinical trials investigating the application of anti-TNF therapies for the treatment of multiple sclerosis, stroke, and Parkinson's and Alzheimer's disease exemplify the excellence of anti-TNF therapeutics as neuroprotective agents [65, 66]. Accordingly, anti-TNF therapy yielded promising results in various retinal degenerative disorders, including glaucoma, retinal ischemia, age-related macular degeneration, and retinitis pigmentosa [63]. Nevertheless, due to its pleiotropic effects in the CNS, inhibition of TNF has been associated with many side effects, which have been proposed to resolve upon selective targeting of sTNF. Given its suspected activity as TNF sheddase, MMP-2 might thus be an interesting target to interfere with sTNF production. Indeed, the detrimental roles of MMPs in various neurodegenerative and (neuro)inflammatory diseases, combined with their druggability, have made them attractive therapeutic targets, and selective MMP-2 inhibition could hence be an alternative for current TACE inhibitors [63, 65, 67].

## Figures and Tables

**Table 1:** Quantification of RGC survival in wild type and MMP-2 null (MMP-2^−/−^) mice, after intravitreal injection of NMDA (1 dpi). RGC densities (RGCs/mm²) per retina are depicted. *N*, number of animals.

	Experiment I				Experiment II			
	Naive		20 mM NMDA		Naive		4.0 mM NMDA	
	Wild type	MMP-2^−/−^	Wild type	MMP-2^−/−^	Wild type	MMP-2^−/−^	Wild type	MMP-2^−/−^
	3243	2960	1428	3110	3267	3228	3019	2931
	3761	3773	1207	2723	3471	3225	3038	3009
	3087	3465	1759	2955	2990	3294	2806	3418
	3428	3687	2830	2836	3465	3354	2759	2927
	3455	3417	2004	3141	3439	3557	2379	2827
		2843	2576		3646	3416	2787	3458
							3046	3276
							2990	3033

Mean	3380	3781	1967	2953	3380	3346	2853	3110
SEM	144	461	259	79	92	51	79	85
*N*	5	6	6	5	6	6	8	8

**Table 2:** Quantification of RGC survival in wild type, MMP-2 null (MMP-2^−/−^), and MMP-9 null (MMP-9^−/−^) mice, after ONC (4 dpi). RGC densities (RGCs/mm^2^) per retina are depicted. *N*, number of animals.

	Naive			ONC		
	Wild type	MMP-2^−/−^	MMP-9^−/−^	Wild type	MMP-2^−/−^	MMP-9^−/−^
	3393	3059	3551	2317	2327	2342
	3265	2928	3458	2473	2262	2663
	3660	3580	3481	2785	2606	2939
	3309	3810	3932	1952	2154	2695
	3187	3759	1825	2856	2641	3122
	3464	2938	2240	2463	2149	2880
			2192		2230	2663
						3209

Mean	3380	3346	2954	2475	2338	2813
SEM	68	169	316	134	77	99
*N*	6	6	7	6	7	8
